# Whole-genome sequencing analysis to identify antimicrobial resistance regions and virulence factors in *Mycobacterium tuberculosis* isolates from the Amhara Region, Ethiopia

**DOI:** 10.1038/s41598-025-01241-6

**Published:** 2025-05-08

**Authors:** Abebe Tesfaye Gessese, Mebrie Zemene Kinde, Tegegne Eshetu, Bizuayehu Kerisew

**Affiliations:** 1 Department of Biomedical Sciences, College of Veterinary Medicine and Animal Sciences, University of Gondar, P.O. Box 196, Gondar, Ethiopia; 2 Department of Medical Parasitology, School of Biomedical and Laboratory Sciences, College of Medicine and Health Sciences, University of Gondar, P. O. Box: 196, Gondar, Ethiopia; 3 Department of Biology, College of Science, Bahir Dar University, Bahir Dar, Ethiopia

**Keywords:** Drug resistance, *Mycobacterium tuberculosis*, Virulence, Whole genome and Amhara, Computational biology and bioinformatics, Molecular biology

## Abstract

Tuberculosis caused by *Mycobacterium tuberculosis* complex is a significant global health burden, with drug-resistant TB, especially multidrug-resistant TB, causing severe challenges to treatment. In Ethiopia, a high TB-burden country, drug resistance has continued spreading. However, some studies indicate genetic diversity, transmission dynamics, and resistance-conferring mutations by using targeted amplification, there are limited reports of whole genome sequencing analysis to uncover the antimicrobial resistance and virulent genes. Based on that, the objective of this project was to identify antimicrobial resistance regions and characterize virulence factors in *M. tuberculosis* isolates through in silico whole-genome sequence analysis. A FASTQ file of 45 *M. tuberculosis* isolates whole genome sequence was downloaded from the SAR database. Following quality control using FASTQC coupled with MultiQC and trimming with Trimmomatic, de novo assembly was conducted using SPAdes. The Burrows-Wheeler Aligner was used for mapping against the *M. tuberculosis* H37Rv reference genome, followed by variant calling with FreeBayes. In silico spoligotyping was performed using SpoTyping, and drug resistance mutations were identified with TB-Profiler and validated using Mykrobe. Virulence factors were detected through ABRicate and the Virulence Factor Database. STRING was used to network the virulent genes. All statistical analyses were performed using R software. This study revealed the most prevalent TB-lineage in the Amhara region was L4 (58.53%), followed by L3 (34.15%), and L1 (4.88%), and in silico spoligotyping classified 90.24% of the isolates into 12 shared types, with SIT 149 (41.46%) and SIT 21 (14.63%) as the most frequent spoligotypes. Seven major genotypic families were identified, with T3-ETH being the dominant family (48.78%). Drug resistance analysis revealed that 38 isolates (92.7%) were multidrug-resistant, and 1 (2.4%) was pre-extensively drug-resistant. Lineage 4 (59%) and its sub-lineage 4.2.2 (51.3%) show the highest resistance. The most frequent mutations to rifampicin, isoniazid, pyrazinamide, ethambutol, streptomycin, ethionamide, fluoroquinolone, and 2nd-line injectable drugs occurred at *rpoB* Ser450Leu, *katG* Ser315Thr, *pncA* c.-11A > G, *embB* Gly406Ala, *rpsL* Lys43Arg, Lys88Thr, *ethA* Met1, *gyrA* Ala90Val, Asp94Asn, and *rrs* 1401A > G, respectively. Additionally, a mutation at the *mmpR5* gene for bedaquiline and clofazimine resistance occurred in one isolate. A total of 67 virulence genes were identified and 63 of them occurred in all isolates. The high prevalence of MDR-TB and the detection of resistance to both first- and second-line drugs in this study underscore the urgent need for enhanced TB control measures in the Amhara region.

## Introduction

Tuberculosis (TB) caused by species within the closely related *Mycobacterium tuberculosis* complex (MTBC), is an ancient human disease that continues to affect millions annually. MTBC is a group of closely related mycobacterial species that cause TB in humans and animals. The MTBC includes several species that are *Mycobacterium tuberculosis, Mycobacterium bovis, Mycobacterium africanum, Mycobacterium microti, Mycobacterium caprae, Mycobacterium pinnipedii, and Mycobacterium canettii.* The MTBC members share a high degree of genetic similarity but differ in their host range, pathogenicity, and epidemiological characteristics. Approximately 98% of human TB cases are caused by *M. tuberculosis*^1^.

Despite significant advances in diagnostic tools, the availability of effective anti-TB therapy, and extensive global efforts, TB remains a major public health concern worldwide and one of the leading causes of death globally^2^. In 2023, an estimated 10.8 million people contracted TB, with approximately 1.25 million deaths reported. The highest burden of TB cases was recorded in South-East Asia (45%), Africa (24%), and the Western Pacific (17%), with smaller shares in the Eastern Mediterranean (8.6%), the Americas (3.2%), and Europe (2.1%)^3^.

The emergence of rifampicin-resistant TB (RR-TB) and multidrug-resistant TB (MDR-TB), as well as resistance to both rifampicin and isoniazid, is a particular concern. The global burden of MDR-TB was estimated at 400,000 new cases in 2023^3^.

Ethiopia is among the 30 high TB-burden countries globally^3^. There were an estimated 143,000 TB cases in the country in 2021 and an estimated 21,000 people died from TB. Ethiopia reported that 51% of notified individuals with bacteriologically confirmed pulmonary TB were tested for rifampicin resistance (RR-TB)^4^. The national five-year TB strategic plan (NSP) was revised in 2020, covering the period from July 2021 to June 2026. Within the NSP period, the Ministry of Health (MOH) targets to reduce TB incidence and mortality from 151 per 100,000 population and 22 per 100,000 population, respectively, in 2018 to 91 per 100,000 population and 7 per 100,000 population, by the end of the NSP^5^.

Drug-resistant tuberculosis (DR-TB) strains, particularly MDR-TB, continue to pose a serious threat to public healthcare systems, mainly in resource-constrained nations such as Ethiopia, where innovative molecular diagnostic technologies and well-equipped laboratory settings are lacking^6,7^. In Ethiopia, the factors contributing to this rise are not fully understood, but genetic differences among mycobacterial strain lineages, which may contribute to resistance-conferring mutations^8,9^, coupled with challenges such as population crowding, the HIV/AIDS epidemic, and poor treatment adherence^10^, play significant roles.

DR-TB usually occurs due to the patient’s delay in early diagnosis and treatment, previous anti-TB drug exposure, inappropriate drug regimens, the patient’s poor adherence to anti-tuberculosis drug regimens, and primary infection with DR-TB strains^11^. Drug resistance in *Mycobacterium tuberculosis* is not a product of a single homogeneous genetic unit. Rather it is a result of frequent mutation in various genes that encode for resistance to antibiotics^12^.

The human-adapted members of MTBC (*M. tuberculosis* and *M. africanum*) are classified into nine lineages with distinct geographic structures^13^. Indo-Oceanic (Lineage 1), East-Asian (Lineage 2), East-African-Indian (Lineage 3), Euro-American (Lineage 4), West-Africa 1 (Lineage 5), West-Africa 2 (Lineage 6), Ethiopian (Lineage 7)^14^, Lineage 8^15^ and Lineage 9^13^ were reported from the Central and Eastern Africa regions, respectively. Among these, the most common lineage on the planet is lineage 4 (L4)^16^.

There are first-line and second-line drugs to treat TB. First-line drugs are the most effective and are used as the initial treatment for drug-sensitive TB. These include Isoniazid (INH), Rifampicin (RIF), Ethambutol (EMB), Pyrazinamide (PZA), and Streptomycin (SM). These drugs are typically used in combination to prevent the development of drug resistance. Second-line drugs are used when TB is resistant to first-line drugs (MDR-TB) or when patients cannot tolerate the first-line drugs due to side effects. These include Fluoroquinolones (Levofloxacin, Moxifloxacin), Injectable agents (Amikacin, Kanamycin, Capreomycin), oral bacteriostatic agents (Ethionamide, Cycloserine, Terizidone), Bedaquiline, Delamanid, Linezolid and Clofazimine^2,17^.

TB drug resistance is classified into five categories. Mono-resistant TB is caused by TB bacteria that are resistant to one first-line anti-TB drug only. Poly-resistant TB is caused by TB bacteria that are resistant to resistance to more than one first-line anti-TB drug, other than both isoniazid and rifampicin. Multidrug-resistant TB (MDR TB) is caused by TB bacteria that are resistant to at least both isoniazid and rifampicin, the most effective first-line TB treatment drugs. Pre-extensively drug-resistant TB (pre-XDR TB) is a type of MDR TB caused by TB bacteria that are resistant to fluoroquinolones in addition to multidrug resistance. Extensively drug-resistant TB (XDR TB) is TB caused by *M. tuberculosis* strains that fulfill the definition of MDR/RR-TB and are also resistant to any fluoroquinolone and at least one additional Group A drug^18,19^.

The WHO has listed more than 30,000 variants of MTBC, detailing their frequency and associations with resistance or susceptibility. This includes identified mutations and summaries of key findings for 13 anti-TB drugs, based on the analysis of over 52,000 isolates with matched whole-genome sequencing and phenotypic drug susceptibility testing data from 67 countries^20^.

Rifampicin-resistant *M. tuberculosis* isolates have mutations in the 81-bp "hot-spot region" of the *rpoB* gene, spanning codons 507–533. The most common mutations occur in codons 516, 526, and 531. Mutations in codons like 518 or 529 are linked to low-level rifampicin resistance but remain susceptible to other rifamycins, such as rifabutin or rifalazil. Nearly all rifampicin-resistant strains are also resistant to other drugs, particularly isoniazid, making rifampicin resistance a key marker for MDR-TB^21^.

Resistance to isoniazid is mainly due to mutations in *katG, inhA, ahpC, kasA, mshA,* and *NDH*. Among these, the most common mutations are S315T in *katG* and 15C/T in *inhA*. Mutations in dfrA may also contribute to resistance, while mutations in the ahpC promoter can serve as markers for resistance^22^. Resistance to ethambutol is primarily linked to mutations in the *embB* gene, codons 306, 406, and 497^23–25^.

Mutations including large deletions in the *pncA* gene are the most common in pyrazinamide-resistant strains. These mutations are spread throughout the gene, primarily within a 561-bp region of the open reading frame or an 82-bp region of the putative promoter^26^.

Mutations in *rpsL* and *rrs* are the main mechanisms of streptomycin resistance. In *rpsL***,** a common mutation is a lysine to arginine substitution at codon 43. In *rrs*, mutations frequently occur near nucleotides 530 and 915. Additionally, mutations in *gidB*, which encodes a 7-methylguanosine methyltransferase for 16S rRNA, result in low-level resistance to streptomycin^27^.

Fluoroquinolone resistance in *M. tuberculosis* is primarily associated with mutations in the *gyrA* and *gyrB* genes, which encode the subunits of DNA gyrase, a critical enzyme in DNA replication. Mutations in *gyrA* and *gyrB* disrupt the action of fluoroquinolones like ofloxacin, levofloxacin, moxifloxacin, and ciprofloxacin, leading to resistance^28^.

Virulent genes of *M. tuberculosis* (MTB) are crucial for the bacteria’s ability to cause disease. These genes help MTB evade the host immune system, survive within host cells, and cause damage to host tissues. Some well-known virulence genes and factors in MTB are ESX-1 Secretion System (esp and esx genes), PPE and PE-PGRS gene families, PhoP-PhoR regulatory system, *KatG* (Catalase-Peroxidase), mce Operons (mce1 and mce4), PknG (Protein Kinase G), LpqH (19-kDa Lipoprotein), HbhA (Heparin-Binding Hemagglutinin Adhesin), Mas and pks Genes (Polyketide Synthases) and Rv1411c (VirS)^29,30^.

According to Comas et al. (2015), L4 predominates across Ethiopia, L3 is widespread but more common in the north, and L7 is mostly found in the northern Ethiopian highlands. Numerous studies have identified spoligotypes SIT 149 and SIT 53 as major clades circulating in Ethiopia (Merid et al., 2021).

In different parts of Ethiopia, few studies have focused on whole-genome sequencing (WGS) of MTB isolates to analyze their genetic diversity, transmission dynamics, and drug resistance patterns. Studies in Northwest Ethiopia and the Tigray region reported L 4 was predominant followed by L3 and L1, L2 and L7 occurred rarely^24,31^. From *Mycobacterium tuberculosis* isolates from the central, eastern, and southeastern Ethiopia six major lineages L4, L3, L2, L1, L5, L6, and L7 were identified^32^. CAS was the most frequent sub-lineage in the Tigray region, followed by Ural and Haarlem^24^. Similarly, Delhi-CAS and EA.ETH (L4.2.2) were the predominant sub-lineages in Northwest Ethiopia^31^. In-silico whole genome sequence analysis of *Mycobacterium tuberculosis* sub-lineage 4.2.2/SIT149 as Dominant Drug-Resistant Clade in Northwest Ethiopia 2020–2022 that showed that L4.2.2.ETH was the leading drug-resistant sub-lineage showed extensive mutations against first-line anti-TB drugs. Ser450Leu/(tcg/tTg) for Rifampicin, Ser315Thr/(agc/aCc) for Isoniazid, Met306Ile/(atg/atA(C)) for Ethambutol, and Gly69Asp for Streptomycin^33^.

Drug-resistant tuberculosis poses significant challenges due to the increased difficulty and cost of treatment. Antimicrobial resistance in TB leads to heightened risks of disease transmission, more severe illness, disability, prolonged and complex treatment regimens, higher healthcare costs, and increased mortality rates.

Several methods have been used in previous studies to investigate resistance genes in *Mycobacterium tuberculosis* (MTB) strains. These methods include targeted gene sequencing of known resistance markers, such as the *rpoB* gene for rifampicin resistance, and the *katG* and *inhA* genes for isoniazid resistance, as well as molecular assays like the GeneXpert MTB/RIF test. Phenotypic drug susceptibility testing (DST) has also been widely employed to determine the resistance profiles of clinical isolates. While effective, these methods may fail to detect novel mutations and genetic factors contributing to drug resistance.

Whole-genome sequencing (WGS) offers a more comprehensive approach by providing detailed insights into both known and novel mutations associated with drug resistance. Although studies in Ethiopia have begun to explore the molecular epidemiology of MTB using WGS, the primary focus has been on lineage distribution and transmission dynamics. Comprehensive studies targeting specific virulence factors and antimicrobial resistance (AMR) regions in *M. tuberculosis* remained limited.

In different parts of Ethiopia, researchers reported the presence of diverse *M. tuberculosis* genotypes using WGS analysis. In Tigray region L4 was predominant, followed by L3. The most frequent mutations to RIF, INH, EMB, SM, PZA, ETH, FLQs, and 2nd-line injectable drugs were also reported^24^. Another comparative whole-genome sequence analysis of *Mycobacterium tuberculosis* isolated from pulmonary tuberculosis and tuberculous lymphadenitis patients in Northwest Ethiopia revealed L4 followed by L3 and then L7^31^. Whole-genome sequencing-based analysis of Mycobacterium tuberculosis isolates from extrapulmonary tuberculosis patients in western Ethiopia showed that the majority of the isolates belonged to Lineage 4 (L4), with L4.6.3 and L4.2.2.2 emerging as the predominant sub-lineages^34^.

This study aimed to address this gap by identifying virulence factors and AMR regions in MTB using WGS data from previously published works on the molecular epidemiology and transmission dynamics of MDR-TB strains in the Amhara region of Ethiopia. Identification of these virulence genes and AMR regions is crucial for addressing the public health threat posed by MDR-TB, as WGS provides a detailed understanding of the genetic mutations responsible for resistance to both first-line and second-line anti-TB drugs.

A deeper understanding of the AMR-conferring mutations in MTB, as well as the associated molecular processes, is essential for improving rapid detection methods, discovering new drug targets, and developing more effective treatments and vaccines to reduce the global burden of TB^24,35^.

Based on the above facts, this study aimed to identify antimicrobial resistance gene mutations and characterize the virulent genes from *M. tuberculosis* isolates in the Amhara region, Ethiopia.

## Methodology

### Whole genome sequence data source

The present study used raw whole-genome sequence data of *M. tuberculosis* in the Amhara region which is available online from an open-source database. A FASTQ file of *M. tuberculosis* whole genome sequence was downloaded from SRA database. The WGS data were from 45 isolates, and the sequencing was performed using a NextSeq 550 desktop sequencer (Illumina, San Diego, CA, USA) to study the molecular epidemiology and transmission dynamics of MDR-TB strains in the Amhara region, Ethiopia by Shibabaw et al. (2023). Genomic DNA from individual strains was prepared for sequencing using Illumina Nextera XT library preparation kits according to the manufacturer’s instructions (Illumina, San Diego, CA, USA). Each isolate was sequenced using paired-end sequencing with 4 replicates for each fastq read file, resulting in two sequencing read files (designated by R1 and R2). The raw sequence data are publicly available under the project accession number PRJNA935744 (https://www.ncbi.nlm.nih.gov/sra/PRJNA935744).

The selection of isolates for WGS analysis was originally based on multiple criteria to ensure a representative and meaningful dataset. The selection criteria were based on the patient’s admission/ data collection year, molecular drug resistance patterns, MDR/RR-TB treatment center hospitals, geographical location of patients, and Lowenstein-Jensen (LJ) culture-positive isolates. A total of 45 isolates were included for WGS analysis. Demographic and clinical characteristics of patients showed that among 45 study participants, the median age was 29 years^25–37^, 60% were male and 20% were HIV-positive. Most of the study participants (67%) had a previous history of anti-TB treatment, the literacy rate was 64% and 51% were urban dwellers. In addition, 18% had a history of contact with MDR/RR-TB patients, 18% had a family history of TB and 91% were smear-positive at the time of diagnosis. The samples were collected from MDR/RR-TB treatment center hospitals in Amhara region, University of Gondar Hospital 19 (42.2%), Boru Meda Hospital 6 (13.3%), Woldia Hospital 6 (13.3%), Ataye District Hospital 5 (11.1%), Finote Selam Hospital 2 (4.4%), Metemma Hospital 3 (6.7%), Debre Birhan Hospital 2 (4.4$), Debre Tabor Hospital 1 (2.2%) and Debre Markos Hospital 1 (2.2%)^36^.

### Sequence read quality check and de novo assembly

The quality assessment of WGS data was checked using FastQC v0.11.9^37^ in aggregation with the MultiQC v1.24.1^38^. Paired-end short reads were trimmed for quality using a flexible read trimming tool for Illumina NGS data Trimmomatic v.0.36 software (sliding-window trimming with a window size of 4 and a read quality threshold of 30), adapter, and other Illumina-specific sequences^39^. The reads were assembled using SPAdes v3.13.0^40^ and the quality of genome assembly was assessed using QUAST v5.2.0 software^41^. WGS of Isolates with coverage less than 30X were considered poor coverage^42^.

### Mapping and variant calling

Mapping was done using Burrows-Wheeler Aligner-Maximal Exact Match (BWA-MEM) algorithm using the *Mycobacterium tuberculosis* H37Rv reference genome. Sequence Alignment/Map tools (SAM tools)^43^ were used for sorting, indexing, removing putative PCR duplicates, and removing temporary files. Then, variant calling was performed using FreeBayes v0.9.21^44^. VCF tools v0.1.16^45^ were used to extract INDELs and SNPs from a VCF file generated from variant calling. The counting of SNPs and Indels from a VCF (Variant Call Format) file was performed using Python, pandas, and seaborn in a Jupyter notebook.

### Spoligotyping

While WGS provides precise mutation data, spoligotyping enhances lineage-level insights, historical comparisons, and epidemiological tracking*. *In silico spoligotyping was conducted using the SpoTyping program version v2.0^46^ with default parameters. The SITVIT2 server was then utilized, based on the identified spoligotypes, to determine the lineage^14^. Isolates exhibiting a similar pattern to those in the SITVIT database were assigned a Spoligo International Type (SIT) number. Isolates that did not match any SIT numbers were categorized as “Orphan” spoligotypes.

### Lineage typing and antimicrobial resistance gene identification

To identify the MTB lineages, sub-lineages, and drug resistance mutations (SNPs, indels, and frameshifts), the isolated strains were analyzed using TB-Profiler v6.2.2. By default, it uses Trimmomatic to trim the reads, BWA to align to the reference genome, and GATK (open source v4) to call variants. This involved aligning raw paired-end illumine sequenced reads against the reference genome MTB H37Rv. To predict resistance the tool uses the curated tbdb database (Phelan et al., 2019). The resistance mutations predicted by TB-Profiler were further validated using mykrobe (v0.10.0)^48^, which provides a list of mutations in genes associated with antimicrobial resistance for each processed strain. Using Mykrobe to validate drug resistance (DR) mutations identified by TBProfiler is crucial due to its independent algorithmic approach, which relies on k-mer detection rather than traditional variant calling. This allows Mykrobe to detect mutations even in poorly mapped or complex genomic regions, potentially identifying low-frequency or novel resistance mutations that TBProfiler may miss. The inclusion of multiple tools for validation enhances the reliability of the results.

### Identification of virulence factors

The tool ABRicate v1.0.1 was used to identify the virulence factor genes of MTB in the Virulence Factor Database (VFDB)^49,50^ of which the threshold for virulence-gene identification using the VFDB was set at a minimum of 80% coverage and identity. The network analysis of the identified virulent gene’s interaction was conducted using STRING v12.0. A High confidence level (0.700) was used as a minimum required interaction score. The network was downloaded in TSV format and visualized using Cytoscape v3.10.2. Markov Clustering (MCL) was used to identify clusters of functionally related proteins. PlasmidFinder-2.0 Server of the Center for Genomic Epidemiology was used to identify plasmids in the assembled sequence of *M. tuberculosis*.

### Statistical analysis

Data were entered using Microsoft Excel, saved as CSV, and imported into R version 4.4.1 for analysis. Data completeness and consistency were assessed by checking for missing values and running frequency distributions for each variable. Descriptive statistics, including frequency and percentage calculations, were conducted as part of the analysis.

## Results

### Whole genome sequence data quality

A FASTQ file of *M. tuberculosis* WGS downloaded from the SAR database was subjected to FASTQC and MultiQc to check its quality. All isolates had mean phred quality > 30. The average read length varied between 227 and 292 bp (mean 282), while the median read length ranged from 127 to 150 bp (mean 149). The mean and median sequencing coverage were 91 and 88 times, respectively.

Out of the 45 sequences three had poor coverage (accession numbers SRR23497702, SRR23497703, and SRR23497959) and one isolate was not identified as *M. tuberculosis* (SRR23497700*)*. These four isolates were excluded and WGS from 41 isolates were used for the downstream analysis. A total of 4291 SNP sites were identified. There were also 26 Indels (8 insertions and 18 deletions).

### Lineage and sub-lineages of *M. tuberculosis* in Amhara Region

**Fig. 1:**
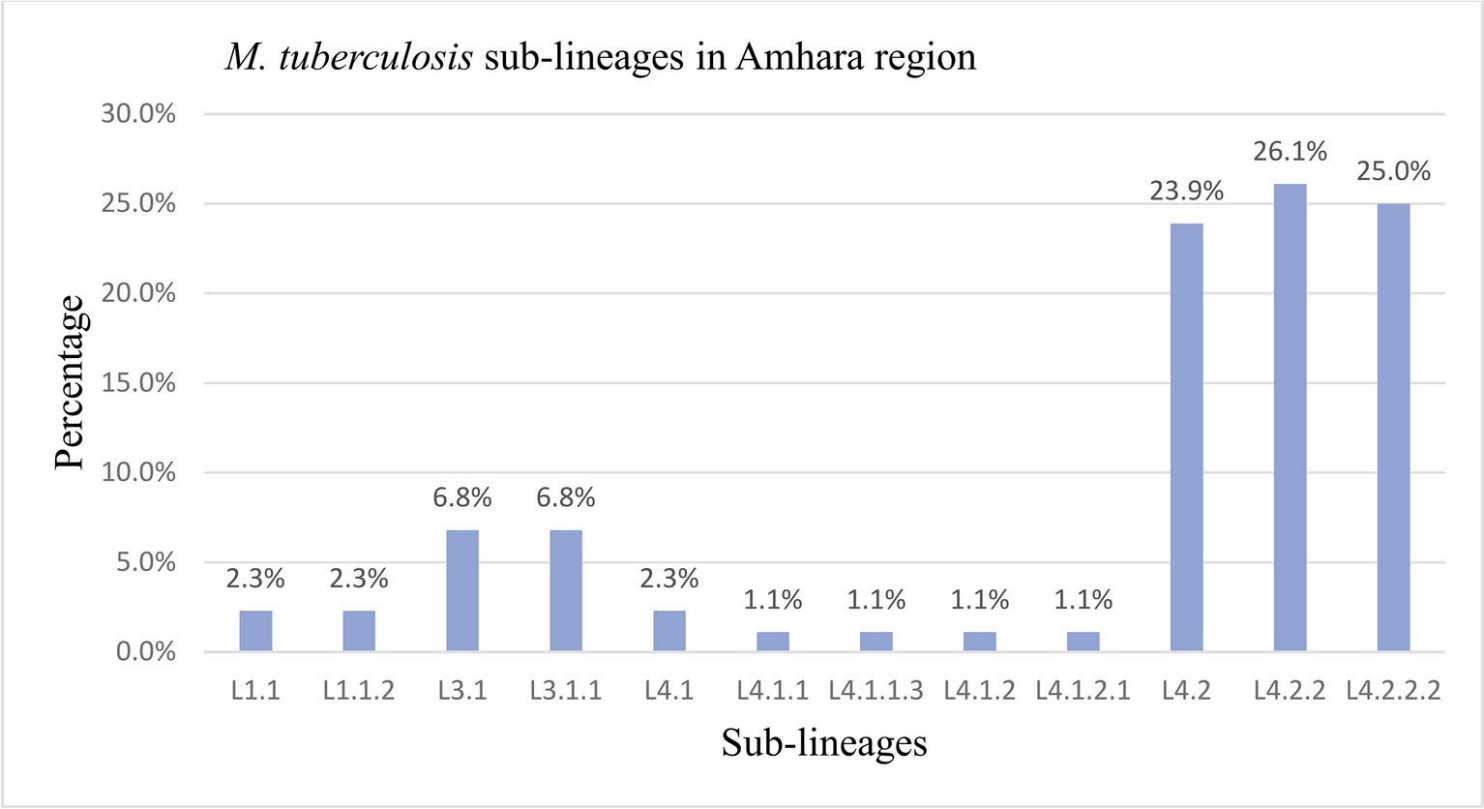
The distribution of *M. tuberculosis* sub-lineages in the Amhara region, Ethiopia (n = 41).

The most frequent lineage was L4 (58.53%), followed by L3 (34.15%), *and* L1 (4.88%) (Table 1). The most common sub-lineages identified were L4.2.2 (26.1%) followed by L4.2.2.2 (25%) and L4.2 (23.9%) (Fig. 1).

**Table 1:** Lineages of *M. tuberculosis* isolates from the Amhara region, Ethiopia (n = 41).

Lineage	n (%)
L1	2 (4.88)
L3	15 (36.59)
L4	24 (58.53)
Total	41(100)

### In silico spoligotyping

According to the spoligotyping findings, 90.24% (37 out of 41) of the isolates were classified into 12 shared types (SIT numbers), while the remaining four isolates (9.76%) were categorized as orphans. Among L3 strains, the most prevalent spoligotype was SIT 25 and SIT 21, while among L4 strains, SIT 149 was the dominant spoligotype (Table 2).

**Table 2:** Spoligotype patterns of *M. tuberculosis* isolates (n = 41) of pulmonary tuberculosis patients in Amhara region, Ethiopia.

Octal code	Binary code	SIT	Main lineages	Family	n(%)
703377400001771	■■■□□□□■■□■■■■■■■■■□□□□□□□□□□□□□□□□■■■■■■■■	21	East-African-Indian	CAS1-Kili	6(14.63)
703777740003731	■■■□□□□■■■■■■■■■■■■■■■□□□□□□□□□□□□■■■■■□■■■	429	East-African-Indian	CAS1-Delhi	1(2.44)
703777740003171	■■■□□□□■■■■■■■■■■■■■■■□□□□□□□□□□□□■■□□■■■■■	25	East-African-Indian	CAS1-Delhi	6(14.63)
777000377760771	■■■■■■■■■□□□□□□□□□□■■■■■■■■■■■■■□□□□■■■■■■■	149	Euro-American	T3-ETH	17(41.46)
777000337760771	■■■■■■■■■□□□□□□□□□□■■□■■■■■■■■■■□□□□■■■■■■■	3362	Euro-American	T3-ETH	1(2.44)
477777376413771	■□□■■■■■■■■■■■■■■■□■■■■■■■□■□□□□■□■■■■■■■■■	None	Indo-Oceanic	None	1(2.44)
777000377740771	■■■■■■■■■□□□□□□□□□□■■■■■■■■■■■■□□□□□■■■■■■■	None	Euro-American	None	1(2.44)
577000377760771	■□■■■■■■■□□□□□□□□□□■■■■■■■■■■■■■□□□□■■■■■■■	Orphan	Euro-American	T3-ETH	1(2.44)
777776777760771	■■■■■■■■■■■■■■■■■□■■■■■■■■■■■■■■□□□□■■■■■■■	119	Euro-American	X1	1(2.44)
074000127540171	□□□■■■■□□□□□□□□□□□□□■□■□■■■■□■■□□□□□□□■■■■■	None	Euro-American	None	1(2.44)
777777777760771	■■■■■■■■■■■■■■■■■■■■■■■■■■■■■■■■□□□□■■■■■■■	53	Euro-American	T1	1(2.44)
701777740003771	■■■□□□□□■■■■■■■■■■■■■■□□□□□□□□□□□□■■■■■■■■■	1551	East-African-Indian	CAS1-Delhi	1(2.44)
477777777413071	■□□■■■■■■■■■■■■■■■■■■■■■■■■■□□□□■□■■□□□■■■■	11	Indo-Oceanic	EAI3-IND	1(2.44)
777000337760771	■■■■■■■■■□□□□□□□□□□■■□■■■■■■■■■■□□□□■■■■■■■	3362	Euro-American	T3-ETH	1(2.44)
777777377760771	■■■■■■■■■■■■■■■■■■□■■■■■■■■■■■■■□□□□■■■■■■■	40	Euro-American	T4	1(2.44)

Moreover, the SITVIT analysis facilitated the identification of seven major genotypic families, with T3-ETH representing the predominant family at 48.78% (20 out of 41), followed by the CAS1-Delhi family comprising 19.51% (8 out of 41) and CAS1-Kili family, comprising 14.63% (6 out of 41) of the isolates. Interestingly, 9.76% (4 out of 41) of the strains corresponded to spoligotypes not previously documented in the SITVIT2 database (Table 2).

### Genetic determinants of drug-resistant tuberculosis

Among 41 *M. tuberculosis* isolates, 38(92.7%; CI 76.9–97.3%) were MDR-TB, one (2.4%; CI 0.1–12.9%) were Pre-XDR-TB and two isolates (4.9%; CI 0.6–16.5%) were susceptible. The highest frequency of drug resistance was recorded in lineage 4 (23; 59%), followed by lineage 3 (14; 35.9%) and lineage 1 (2; 5.1%). Among the sub-lineages of lineage 4, lineage 4.2.2 has the highest frequency of drug resistance, with 20 isolates, making up 51.3% of the total, followed by lineage 4.1 (2; 5.1%) and lineage 4.2 (1; 2.6%).

From the 39 MDR and Pre-XDR-TB isolates, 37 (94.9%) were resistant to rifampicin. All the mutations have occurred at the *rpoB* gene, and the dominant mutation was at codon Ser450Leu (24; 64.9%). There were 37 isolates that showed isoniazid resistance of which, 34 (91.9%) exhibited mutations in the *katG* gene, two (5.4%) in the *inhA* gene, and one (2.7%) in both *katG* and *inhA* genes. Among isolates with mutations in the *katG* gene, 32 (94.12%) had the Ser315Thr mutation, while one had the Ser315Ile mutation. Additionally, one isolate exhibited both Ser315Thr and Ile317Val mutations at genome positions 2,155,168 and 2,155,163, respectively. All mutations in the *inhA* gene were observed at codon c.-777C > T. In one isolate, mutations were found in both *katG* gene codon Ser315Thr and *inhA* codon c.-154G > A alias fabG1p.Leu203Leu. Pyrazinamide resistance was shown in 22 (56.4%) isolates and all the mutations were at the pncA gene. Mutations showed at diverse codons with a higher frequency at codon c.-11A > G.

Resistance-conferring mutations in 31 (79.5%) ethambutol-resistant isolates occurred in the *embB* and *embA* genes. In 27 (87.1%) isolates, resistance mutations were identified at *embB* codons Gly406Ala (11; 40.7%), Met306Ile (6; 22.2%), Gly406Ser (2; 7.4%), Asp328Gly (1; 3.7%), Asp328Tyr (1; 3.7%), and Gln497Arg (1; 3.7%). Double mutations were detected at *embB* codons Met306Ile and Asp1024Asn (1; 3.7%), as well as Met306Val and Gln497Arg (1; 3.7%). In four (12.9%) isolates, mutations occurred in both the *embA* codons c.-16C > T (3; 75%) and c.-12C > T (1; 25%), as well as in the *embB* codon Met306Ile.

Mutations of streptomycin resistance were identified in 34 isolates. Mutations in the *rpsL* gene occurred in 10 isolates (29.4%) at codon Lys43Arg (8; 80%), Lys88Thr (1; 10%), and Lys88Gln (1; 10%). Mutations were also observed in both the *rpsL* and gid genes in 10 isolates (29.4%), involving codon Lys88Thr in *rpsL* and Gly69Asp in gid. Additionally, in 9 isolates (26.5%), resistance mutations were found at the gid codon Gly69Asp. Moreover, streptomycin resistance mutations were observed in the rrs gene in 2 isolates (5.9%) at codon n.888G > A, as well as in both the rrs and gid genes in 2 isolates (5.9%) at codons 799C > T and Gly69Asp, respectively. In 1 isolate (2.94%), mutations were identified in all three genes *rpsL*, rrs, and gid at codons Lys88Thr, 799C > T, and Gly69Asp, respectively.

Ethionamide resistance was observed in 27 isolates (69.2%), with mutations in the *ethA* gene found in 23 isolates (85.2%). Of these, a mutation at codon p.Met1? was identified in 17 isolates (73.9%), while the c.859_999del mutation appeared in 2 isolates (8.7%). Additionally, mutations in the *inhA* gene were present in 4 isolates (14.8%), with the c.-777C > T mutation detected in 3 isolates (75%) and the c.-154G > A mutation, also known as fabG1 Leu203Leu, found in one isolate. The drug resistance-conferring mutation for 2nd-line anti-TB drugs occurred at *gyrA* codon Ala90Val and Asp94Asn for fluoroquinolone (levofloxacin and moxifloxacin) resistance in one isolate and rrs gene codon 1401A > G, 1402C > A, 1484G > T, noncoding transcript exon variant, for 2nd-line injectable drugs amikacin, kanamycin, and capreomycin in four isolate for each. Additionally, a mutation at mmpR5 gene codon c.338_339insC and c.273_274insG, frameshift mutation, for bedaquiline and clofazimine resistance, respectively in one isolate (Table 3).

**Table 3:** Drug resistance pattern and associated resistance-conferring mutation of M. tuberculosis in Amhara region, Ethiopia (n = 39).

Drug	Gene	Genome position	Mutations	Variant type	n (%)
Rifampicin	*rpoB*	761,095	p.Leu430Pro	Missense variant	37(94.9)
		761,109	p.Asp435Tyr		
		761,110	p.Asp435Val		
		761,113	p.Gln436Pro		
		761,114	p.Gln436His		
		761,115	p.Asn437Tyr		
		761,139	p.His445Cys		
			p.His445Tyr		
		761,140	p.His445Arg		
		761,155	p.Ser450Leu		
		761,161	p.Leu452Pro		
		761,277	p.Ile491Phe		
Isoniazid	*katG*	2,155,168	p.Ser315Thr	Missense variant	37(94.9)
		2,155,163	p.Ile317Val		
		2,155,167	p.Ser315Thr		
		2,155,628	p.Ala162Thr		
		1,674,263	p.Ile21Thr		
		1,673,425	c.-777C > T	Upstream gene variant	
	*inhA*	2,155,168	p.Ser315Thr	Missense variant	
	*katG and inhA*	1,674,048	c.-154G > A Alias fabG1_p.Leu203Leu	Upstream gene variant	
Pyrazinamide	*pncA*	2,289,252	c.-11A > G	Upstream gene variant	22(56.4%)
		2,288,885	p.Trp119Cys	Missense variant	
		2,289,016	p.Thr76Pro		
		2,288,722	p.Glu174*	Stop gained	
		2,289,227	c.-63_14delATCT…	Frameshift variant & start lost	
		2,284,911	c.474_*3769del	Frameshift variant & stop lost & splice region variant	
		2,289,049	c.192_193insA	Frameshift variant	
		2,288,952	p.Gly97Val	Missense variant	
Ethambutol	*embA*	4,243,221	c.-12C > T	Upstream gene variant	31(79.5%)
		4,243,217	c.-16C > T		
	*embB*	4,247,429	p.Gly406Ser	Missense variant	
		4,247,430	p.Gly406Ala		
		4,247,431	p.Met306Ile		
		4,247,496	p.Asp328Gly		
		4,247,495	p.Asp328Tyr		
		4,248,003	p.Gln497Arg		
		4,249,583	p.Asp1024Asn		
		4,247,429	p.Met306Val		
Streptomycin	*gid*	4,407,997	p.Gly69Asp	Missense variant	34(87.2%)
	*rpsL*	781,822	p.Lys88Thr		
		781,687	p.Lys43Arg		
	*rrs*	1,472,733	n.888G > A	Noncoding transcript exon variant	
		1,472,644	n.799C > T		
Amikacin	*rrs*	1,473,246	n.1401A > G	Noncoding transcript exon variant	4 (10.3)
		1,473,247	n.1402C > A		
		1,473,329	n.1484G > T		
Bedaquiline	*mmpR5*	779,327	c.338_339insC	Frameshift variant	1(2.6)
Capreomycin	*rrs*	1,473,246	n.1401A > G	Noncoding transcript exon variant	4 (10.3)
		1,473,247	n.1402C > A		
		1,473,329	n.1484G > T		
Clofazimine	*mmpR5*	779,262	c.273_274insG	Frameshift variant	1(2.6)
Ethionamide	*ethA*	4,327,472	p.Met1?	Start lost	27(69.2)
		4,326,474	c.859_999del	Conservative inframe deletion	
		4,326,183	c.1290delC	Frameshift variant	
		4,326,461	p.Ile338Ser	Missense variant	
		4,326,066	c.1407delG	Frameshift variant	
	*inhA*	1,674,048	c.-154G > A	Upstream gene variant	
		1,673,425	c.-777C > T		
		1,674,263	p.Ile21Thr	Missense variant	
		1,674,048	c.-154G > A Alias fabG1_p.Leu203Leu	Upstream gene variant	
Kanamycin	*rrs*	1,473,246	n.1401A > G	Noncoding transcript exon variant	4 (10.3)
		1,473,247	n.1402C > A		
		1,473,329	n.1484G > T		
Levofloxacin	*gyrA*	7570	p.Ala90Val	Missense variant	1(2.6)
		7581	p.Asp94Asn		
Moxifloxacin	*gyrA*	7570	p.Ala90Val	Missense variant	1(2.6)
		7581	p.Asp94Asn		

Varying levels of drug resistance across different sublineages were found. The most common form of drug resistance observed is MDR-TB, which is present in all sublineages, with the highest frequency found in sublineages L 4.2**,** L 4.2.2, and L 4.2.2.2**,** each exhibiting 21 cases. Pre-XDR TB was detected in sublineages L 3.1 and L 3.1.1, each showing one case. Mono-resistant TB (streptomycin resistance) was found in L 3, with one case (Table 4).

**Table 4:** The type of drug resistance in each sublineage of *M. tuberculosis* in Amhara region, Ethiopia (n = 41).

Sublineage	Type of drug resistance	Frequency
L 1.1	MDR-TB	2
L 1.1.2	MDR-TB	2
L 3.1	MDR-TB	5
	Pre-XDR TB	1
L 3.1.1	MDR-TB	6
	Pre-XDR TB	1
L 3	MDR-TB	6
	Monoresistant TB	1
L 4.1	MDR-TB	2
L 4.1.1	MDR-TB	1
L 4.1.1.3	MDR-TB	1
L 4.1.2	MDR-TB	1
L 4.2	MDR-TB	21
L 4.2.2	MDR-TB	21
L 4.2.2.2	MDR-TB	21

### Virulence genes of* Mycobacterium tuberculosis* isolates

A total of 66 virulence genes were identified, with 63 virulent genes (95.45%) found in all isolates. The identified genes had a coverage range of 81.4% to 100% (mean 99.94%) and an identity range of 83.1% to 100% (mean 99.34%) when compared to the virulence factor database. The gene espB was present in 9 isolates, espK in 2 isolates, and eccA1 in 1 isolate (Table 5). Virulent genes esxN and esxM were found frequently in all isolates of *M. tuberculosis* with a total frequency of 126 and 125, respectively.

**Table 5:** Identified virulent genes of *M. tuberculosis* in Amhara region, Ethiopia (n = 41).

Accession number of isolates	Identified virulence genes
SRR23497682, SRR23497683, SRR23497684, SRR23497685, SRR23497686, SRR23497687, SRR23497688, SRR23497689, SRR23497690, SRR23497691, SRR23497692, SRR23497693, SRR23497694, SRR23497695, SRR23497696, SRR23497697, SRR23497698, SRR23497699, SRR234977001, RR23497704, SRR23497705, SRR23497706, SRR23497707, SRR23497708, SRR23497709, SRR23497710, SRR23497711, SRR23497712, SRR23497713, SRR23497714, SRR23497715, SRR23497716, SRR23497717, SRR23497718, SRR23497957, SRR23497958, SRR23497960, SRR23497961, SRR23497962, SRR23497963 and SRR23497964	eccA3, eccA5, eccB1, eccB3, eccB5, eccC3, eccCa1, eccCa5, eccCb1, eccCb5, eccD3, eccD5, eccE1, eccE3, eccE5, erp, espA, espC, espD, espG3, esxA, esxB, fbpA, fbpB, fbpC, hbhA, hspX, ideR, irtA, irtB, lipF, mbtA, mbtB, mbtC, mbtD, mbtE, mbtF, mbtG, mbtH, mbtI, mbtJ, mbtK, mbtL, mbtM, mbtN, mgtC, mycP1, mycP3 mycP5, PE35, PE5, phoP, phoR, PPE4, PPE41, relA, Rv1794, eccD1, esxG, esxH, esxM, esxN, icl
In all except SRR23497685	eccA1, espK
SRR23497682, SRR23497683, SRR23497695, SRR23497699 SRR23497706, SRR23497707, SRR23497714, SRR23497715 SRR23497963	espB
In all except SRR23497717	espK

### Network analysis of identified virulence genes of MTB isolates

**Fig. 2:**
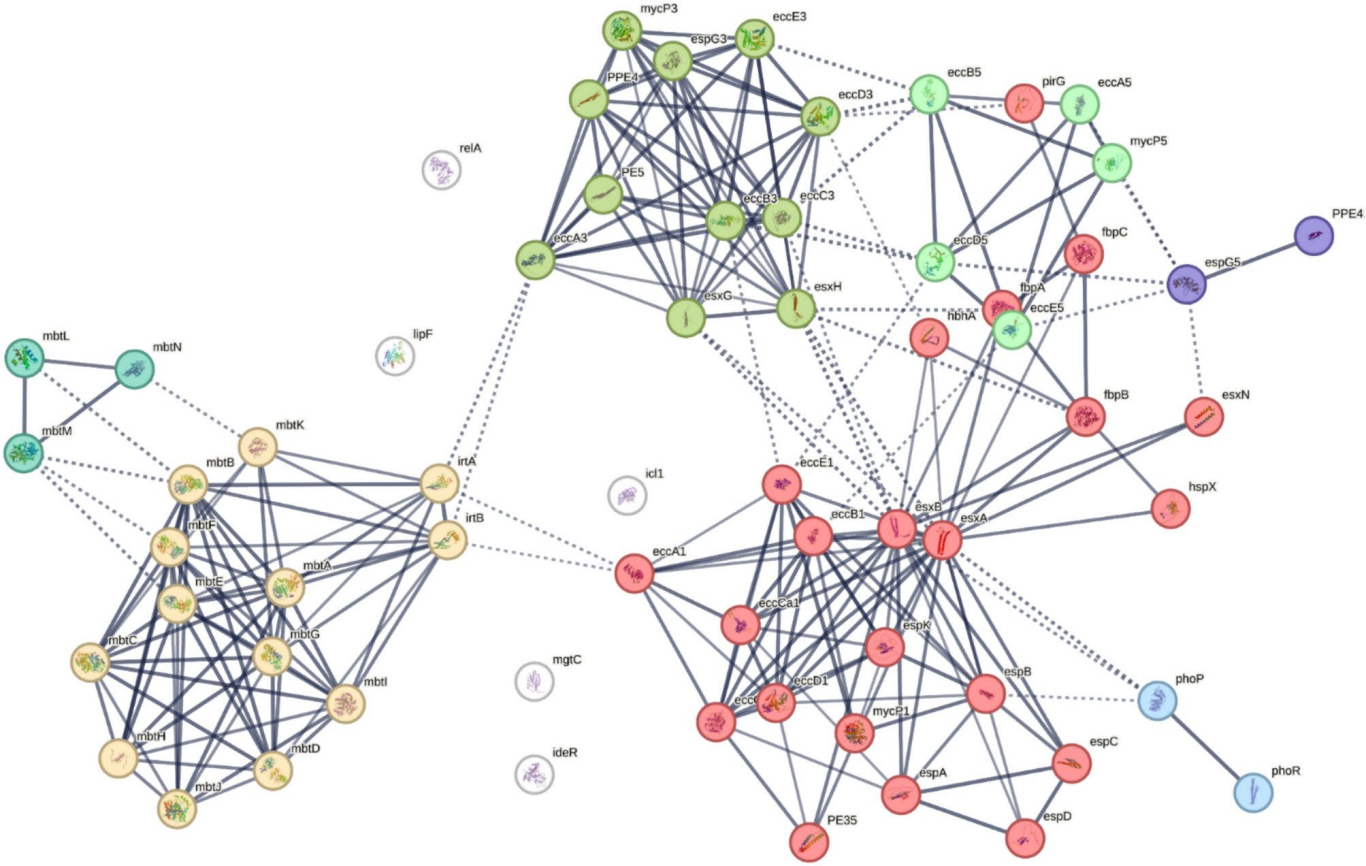
Protein interaction network of virulent genes of MTB isolates in Amhara region, Ethiopia.

In the network analysis, the identified virulent genes were recognized as nodes and there were 242 edges connecting the nodes. The average node degree was 7.68 and the average local clustering coefficient was 0.639. The network has significantly more interactions than expected, such an enrichment indicates that the proteins are at least partially biologically connected, as a group (Fig. 2). There were five isolated genes (relA, lipF, icl1, mgtC, and ideR) from the network.

Markov clustering of the network grouped the genes into seven distinct clusters based on functional classifications. Cluster 1 (Red), 22 genes involved in protein secretion by the type VII secretion system. Cluster 2 (Yellow), 13 genes related to biosynthesis of siderophore group nonribosomal peptides. Cluster 3 (Orange), 11 genes classified as mixed, including organelle lumen and zinc ion homeostasis. In cluster 4 (Green), 5 genes were classified as mixed, including EccD-like transmembrane domain and peptidase S8, subtilisin, and Asp-acti. Cluster 5 (Light green), 3 genes involved in catechol-containing compound metabolism and long-chain fatty acid metabolism. Cluster 6 (Light blue), 2 genes linked to the two-component regulatory system. In cluster 7 (Purple), 2 genes are classified as mixed, including the EspG family and PPE family (C-terminal) (Fig. 2). There was no plasmid found in all isolates.

## Discussion

The current study highlights the genetic diversity, AMR, and virulence genes of MTB strains circulating in the Amhara region of Ethiopia.

Among 41 *M. tuberculosis* isolates 39 had drug resistance mutations. In most discordant cases, isolate pairs harbored variants that could cause low- or moderate-level resistance or were previously associated with variable minimum inhibitory concentrations (MICs)^51^. Additionally, resistance detection through WGS relies on known databases of resistance-conferring mutations, and some isolates may carry novel or rare variants that are not yet well characterized^52^. The simultaneous performance of phenotypic DST and WGS-based resistance profiling provides the most accurate assessment of drug resistance in *M. tuberculosis* isolates^51^.

Among the identified lineages, L4 (58.53%) was the most frequent followed by L3 (36.59%). This finding is consistent with previous studies conducted across various regions of Ethiopia, from Tigray region^24^ Northwest Ethiopia^31^ St.Peter’s TB Specialized Hospital, Ethiopia^53^, central, eastern, and southeastern Ethiopia^32^, and a nationwide review in Ethiopia^54^. Afro-TB dataset of MTB in Africa also showed L4 and L3 were the predominant lineages in Ethiopia^55^. Ethiopia’s location as a crossroads between Africa, the Middle East, and Europe has contributed to the introduction and spread of different MTB lineages. Historically, migration and trade routes likely facilitated the spread of L4 (originating from Europe) and L3 (originating from the Indian subcontinent) into Ethiopia^56^. Nevertheless, a lower proportion (40.1%) of lineage 4 has been documented in Northwest Ethiopia^57^. However, another study conducted among refugees residing in refugee camps in Ethiopia reported that lineage 3 (52, 77.6%) was the prevalent lineage. This could be due to that people in the refugee camps were from different countries (Eritrea, Somalia, Sudan, and South Sudan), which contributed to the dominance of this lineage in the camp^58^.

In terms of sub-lineages, L4.2.2 (26.1%) was the most frequent, followed by L4.2.2.2 (25.0%) and L4.2 (23.9%). Similarly, a study by^31^ reported that L4.2.2 was the predominant sub-lineage in Northwest Ethiopia. The high prevalence of sub-lineages within L4 was also reported by previous studies in different parts of Ethiopia^34,59,60^. However, these previous studies did not report L4.2.2 and L4.2.2.2 in high frequencies as observed in the present study.

Genotypes of lineage 3 of *M. tuberculosis* infection showed a predominance of the CAS1-Dehli family; this agrees with other studies^57,61,62^. The other dominant family was CAS1-Kili, which was also reported previously in Ethiopia^24^ and was not reported in a previous study in Ethiopia^60^. In lineage 4, T3-ETH was the most prevalent. Another study in Northwest Ethiopia also reported that L4-T3-ETH (32.0%), L3-CAS1-Delhi (22.7%), and L3-CAS1-Killi (14.8%) families were the most common^63^. The dominantly identified Spoligo SITs were SIT25, T3-ETH and SIT149. The finding is in agreement with previous reports^64,65^. The highest frequency of drug resistance was recorded in lineage 4 (59%), Among the sub-lineages, sub-lineage 4.2.2 had the highest frequency (51.3%) of drug resistance. This finding is comparable with previous reports in different parts of Ethiopia^33,63^. Prior studies in Ethiopia showed that these spoligotypes were more often linked with drug resistance-conferring mutations and clonal expansion in Ethiopia^66^. Perhaps, the connection of certain MTBC genotypes with MDR could be attributed to their genetics and enhanced intrinsic ability to acquire resistance to anti-TB drugs^67^.

Resistance of MTB strains to RIF is mainly due to canonical mutations in the hot-spot region of the *rpoB* gene (HSRrpoB). However, there are also disputed *rpoB* mutations that confer RIF-resistance, and their occurrence is not rare^68^. The association of these mutations with RIF resistance is endorsed by WHO in the updated catalog of mutations in MTBC and their association with DR^20^. Rifampicin works by binding to this subunit, inhibiting RNA synthesis and effectively killing *Mycobacterium tuberculosis*^69^. Specific mutations on the *rpoB* gene, alter the binding site of rifampicin on RNA polymerase, preventing the drug from binding effectively. This allows the bacteria to continue transcribing DNA into RNA, despite the presence of rifampicin^70,71^.

According to the WGS analysis, 91.9% of mutations that confer resistance to isoniazid occurred at the *katG* codon Ser315Thr, which is the most commonly reported mutation associated with high-level isoniazid resistance^24,72,73^. The *KatG* gene encodes the enzyme catalase-peroxidase and mutation in this gene decreases or blocks the enzyme activity. Mutation in the *katG* gene is the main mechanism of INH resistance in most strains^74^. The *inhA* mutations with or without a *katG* mutation were detected in the present study. There are similar reports in previous studies^75,76^.

In addition to rifampicin and isoniazid resistance, pyrazinamide resistance was detected in 56.4% of the isolates, with mutations in the *pncA* gene being responsible for the resistance. The diversity of mutations observed in the *pncA* gene highlights the complexity of pyrazinamide resistance mechanisms which has been reported in other studies^24,33^. The gene pncA is one such gene that encodes for pyrazinamidase (PZAse) and helps in the activation of pyrazinamide (PZA) to its active form pyrazinoic acid (POA). Thus, a mutation in this gene is linked to PZA resistance^77^.

Ethambutol (EMB) resistance was found in 79.5% of the isolates, with mutations in the *embB* and *embA* genes. The most common mutations of *embB* were at codons 406 and 306, which are considered as hotspot resistance codons^78^. There were also mutations at codons 328 and 497. Previous reports showed that codon 306 was shown to be directly involved in EMB binding while codons 406 and 497 were not directly involved^78,79^. Nevertheless, mutations at codon 497 cause conformational changes that affect codon 327, one of the EMB binding sites. Codon 406 mutations may also affect drug binding by causing protein conformation changes^78^. Mutations at the *embA* gene also confer resistance to ethambutol. This result aligns with the principle that resistance to EMB is caused by mutation of the embCAB operon (*embC*, *embA*, and *embB*) that encodes membrane-associated arabinosyltransferases involved in the synthesis of cell wall arabinogalactan^80^.

In our study, mutations that confer SM resistance were observed at the *rpsL, gid,* and *rrs* genes. Previous studies have indicated that most SM-resistance *Mycobacterium tuberculosis* isolates can be determined by mutations in *rpsL*, rrs, and gid^27^. The most prevalent mutation associated with SM-resistance was at the *rpsL* gene codon Lys43Arg. Although the proportion of mutations that confer resistance to SM at Lys43Arg varied geographically, this finding is concordant with an earlier study that reported its dominance across the world and its association with a high drug resistance level^81^. Similar findings were also reported by^24^. The frequently detected mutation at K43R highlighted its importance as a surrogate marker for rapid detection of SM-resistance. The present findings indicated that 29.4% of isolates showed co-existing mutations at both genes, *rpsL* codon Lys88Thr in *rpsL* and *gid* codon Gly69Asp.

Ethionamide resistance-conferring mutations occurred at *ethA* gene codon M1 (85.2%), *inhA* and *inhA* alias with fabG1 gene (14.8%). The present finding revealed that all isolates resistant to ETH were co-resistant to INH. This finding is supported by prior reports that showed the isolation of Mtb strains co-resistant to INH and ETH from TB patients previously treated with INH but never treated with ETH^24,82^. The fabG1 gene codon C-154G > A mutation, which conferred resistance to both INH and ETH, was detected from all MDR-TB. This is consistent with other study reports^24,83^.

The study also detected resistance-conferring mutations for second-line anti-TB drugs, including fluoroquinolones (levofloxacin and moxifloxacin) and injectable drugs (amikacin, kanamycin, and capreomycin). According to the WHO Consolidated Guidelines (2022), second-line injectable drugs are no longer recommended in the treatment regimen for drug-resistant TB (DR-TB) due to their lower efficacy and higher toxicity^84^. In one isolate mutations in the *gyrA* gene codons Ala90Val and Asp94Asn conferring fluoroquinolone resistance were detected. This finding is supported by previous reports^85^. However, the mutation sites and frequencies of gyrA varied across studies^86–89^, which may be attributed to the difference in detection techniques, breakpoint concentrations, epidemic strains, research population, and medical history. Four isolates had mutations in the *rrs* gene at codons 1401A > G, 1402C > A, and 1484G > T, which are associated with resistance to aminoglycosides and capreomycin. This finding agrees with previous reports^90,91^. These findings underscore the potential emergence of XDR-TB in the region, which could complicate treatment options and outcomes.

The detection of mutations in the mmpR5 gene (Rv0678) has been associated with resistance to bedaquiline and clofazimine, two essential drugs in the treatment of rifampicin-resistant tuberculosis (TB). Previous studies (Nimmo et al., 2024) highlighted similar findings, suggesting that baseline resistance due to mmpR5 mutations increases the risk of treatment failure in an all-oral 6-month regimen that includes bedaquiline, linezolid, and moxifloxacin (Timm et al., 2023). Importantly, some mmpR5 mutations, like Met146Thr, emerged before the introduction of bedaquiline, conferring cross-resistance to both bedaquiline and clofazimine. This mutation is now recognized in the WHO’s updated catalogue of resistance mutations (Beckert et al., 2020; WHO, 2023). The emergence of such resistance poses a challenge for the treatment of multidrug-resistant (MDR) and extensively drug-resistant (XDR) TB, threatening the efficacy of future regimens. It is worth noting that mutations in the ethA, fabG1, and mmpR5 genes cannot be detected by LPA.

Several virulent genes associated with the MTB strain were identified. The *ecc* genes (*eccA, eccB, eccC, eccCa, eccCb, eccD, eccE*) are part of the ESX (Type VII secretion) systems that play an important role in the secretion of virulence factors, which are crucial for the pathogenicity of *M. tuberculosis*^92^*.*

The identified *esp* and *esx* genes (*espA, espB, espC, espD, espG3, espK, esxA, esxB, esxG, esxH, esxN, and esxM*) are part of the Type VII Secretion Systems (T7SS) in *Mycobacterium tuberculosis* and are essential for its virulence. These genes encode proteins that help secrete virulence factors, allowing the bacterium to survive and evade the host immune system. Their identification highlights the high virulence potential of these genes, given their crucial roles in intracellular survival, immune evasion, and nutrient acquisition^93^. About 20.7% of L4 and 33.3% of L3 have only *espB* gene. The ESX-1 system, with key components such as EsxA and EsxB, allows M. tuberculosis to escape from the phagosome and survive inside macrophages. Meanwhile, the ESX-3 system, supported by EsxN and EsxM, enables the bacterium to acquire essential metals (such as iron and zinc) from the host, which is crucial for its growth and persistence. Furthermore, the secretion of proteins like EspA, EspB, and EspC helps M. tuberculosis evade the host immune response, creating a more favorable environment for its replication^93–95^.

The genes fbpA, fbpB, and fbpC in Mycobacterium tuberculosis encode proteins that are part of the antigen 85 complex (Ag85), which plays a crucial role in the virulence and pathogenesis of M. tuberculosis. This complex is composed of three main proteins Ag85A, Ag85B, and Ag85C encoded by *fbpA, fbpB,* and *fbpC*, respectively. These proteins are involved in key biological processes that contribute to the bacterium’s ability to establish infection and persist within the host^96^.

The *mbtA, mbtB, mbtC, mbtD, mbtE, mbtF, mbtG, mbtH, mbtI, mbtJ, mbtK, mbtL, mbtM, and mbtN* genes in *Mycobacterium tuberculosis* comprise the mycobactin biosynthetic gene cluster. Their primary function is to facilitate the synthesis of mycobactins, which are siderophore-like molecules crucial for sequestering iron from the host environment^97^. Iron is vital for bacterial growth and metabolism, particularly during infection. The ability to acquire iron is directly linked to the virulence of *M. tuberculosis*, and the mbt gene cluster is essential for establishing infection and persistence in the host^98^. Understanding the mechanisms of mycobactin biosynthesis and regulation can inform vaccine development and novel treatment strategies against tuberculosis.

There was no plasmid in all isolates and this finding is supported by lacking previous evidence of the presence of plasmids in *M. tuberculosis*^99^*.* These finding is a good insight that *M. tuberculosis* does not rely on plasmids for survival or virulence, but its genome is rich in virulence factors and complex regulatory systems encoded on its chromosome.

### Limitations

While this study provides valuable insights into the genetic diversity, antimicrobial resistance (AMR) patterns, and virulence factors of *M. tuberculosis* strains in the Amhara region, it has limitations. The reliance on whole-genome sequencing (WGS) without phenotypic drug susceptibility testing (DST) limits the validation of resistance profiles. Additionally, the study lacked clinical and epidemiological data, which would have provided a more comprehensive understanding of the factors driving drug-resistant TB spread. Resource constraints also limited the depth of genomic analysis.

## Conclusion and recommendations

This study demonstrated that TB in the Amhara region is caused by a wide diversity of MTB strains belonging to lineages L1, L3, and L4 with a predominance of L4-T3-ETH, L3-CAS1-Delhi and L3-CAS1-Killi families. Overall, L4 was the most frequently observed MTB genotype and was associated with the highest proportion of drug resistance. The study also highlighted the usefulness of mutations at *rpoB, katG, embB, rpsL, pncA, ethA, gyrA, and rrs* genes as molecular markers for the rapid detection of resistance for RIF, INH, EMB, SM, PZA, ETH, FLQs, and SLIDs, respectively. This study also provided valuable insights into the virulence genes of *Mycobacterium tuberculosis* isolates from the Amhara region of Ethiopia. Notably, the virulent genes *esxN* and *esxM* were frequently present in all isolates. The high prevalence of MDR-TB and the detection of resistance to second-line drugs, including bedaquiline and clofazimine, underscore the urgent need for advanced molecular diagnostic tools. Establishing a robust surveillance system to monitor the spread of resistant strains and their genetic evolution can inform public health interventions and prevent the further spread of resistant TB. Tailoring treatment based on the genetic profile of the infecting strain can improve treatment outcomes and reduce the risk of further resistance development.

## Data Availability

All data generated or analyzed during this study are available upon the request of the corresponding author.

## Abbreviations

AMR: Antimicrobial resistance
MTB: *Mycobacterium tuberculosis*
MDR-TB: Multidrug-resistant tuberculosis
FLQ: Fluoroquinolones
SLID: Second line injectable drugs
WGS: Whole genome sequencing
